# Resting-state brain network in Parkinson’s disease with different degrees of depression

**DOI:** 10.3389/fnins.2022.931365

**Published:** 2022-09-21

**Authors:** Qinru Liu, Zhenni Mao, Changlian Tan, Sainan Cai, Qin Shen, Min Wang, Junli Li, Lin Zhang, Fan Zhou, Chendie Song, Jiaying Yuan, Yujing Liu, Jun Liu, Haiyan Liao

**Affiliations:** ^1^Department of Radiology, The Second Xiangya Hospital, Central South University, Changsha, China; ^2^Department of Radiology, The Third Hospital of Changsha, Changsha, China; ^3^Clinical Research Center for Medical Imaging in Hunan Province, Changsha, China

**Keywords:** Parkinson’s disease, depression, independent component analysis, resting-state functional magnetic resonance imaging, functional connectivity

## Abstract

**Objective:**

The aim of this study is to explore the neural network mechanism of Parkinson’s disease (PD) with different degrees of depression using independent component analysis (ICA) of the functional connectivity changes in the forehead, limbic system, and basal ganglia regions.

**Methods:**

A total of 106 patients with PD were divided into three groups: PD with moderate-severe depression (PDMSD, *n* = 42), PD with mild depression (PDMD, *n* = 29), and PD without depression (PDND, *n* = 35). Fifty gender- and age-matched healthy subjects were recruited as a control group (HC). Three-dimensional T1-weighted image and resting-state functional magnetic resonance imaging (RS-fMRI) data were collected.

**Results:**

Different functional connectivity was observed in the left precentral gyrus, right precuneus, right inferior frontal gyrus, right medial and paracingulate gyrus, left supplementary motor area, right brain insula, and the inferior frontal gyrus of the left orbit among the four groups (ANOVA, *P* < 0.05, Voxel size > 5). Both PDMD and PDMSD exhibited increased functional connectivity in the superior-posterior default-mode network (spDMN) and left frontoparietal network (LFPN); they also exhibited a decreased functional connectivity in the interior Salience Network (inSN) when compared with the PDND group. The functional connectivity within the inSN network was decreased in the PDMSD group when compared with the PDMD group (Alphasim correction, *P* < 0.05, voxel size > 5).

**Conclusion:**

PD with different degrees of depression has abnormal functional connectivity in multiple networks, which is an important neurobiological basis for the occurrence and development of depression in PD. The degree of decreased functional connectivity in the inSN network is related to the degree of depression in patients with PD-D, which can be an imaging marker for PD to judge the severity of depression.

## Introduction

Parkinson’s disease (PD) is the second fastest growing neurodegenerative disease and a risk factor for the health and life of middle-aged and elderly people. Depression is one of the common non-motor and psychiatric symptoms of PD (Seppi et al., 2019). The reported incidence of depression in PD (PD-D) ranges from 2.7 to 90%, with an average of 40% (Schrag and Taddei, 2017). Depression is mainly mild to moderate in PD, and only a small number of patients with PD have severe depression (accounting for 2–7%) (D’Ostilio and Garraux, 2016). Depression is easily missed during the diagnosis of PD due to the lack of specific clinical manifestations; depressive symptoms also often overlap with motor symptoms. Therefore, early diagnosis of PD-D is significant for patients.

The rapid development of new neuroimaging technologies has provided a technical guarantee for the early diagnosis of PD-D. Resting-state functional magnetic resonance imaging (RS-fMRI) has become one of the most promising methods to study the neural mechanisms of PD-D. Most of the previous studies from the perspective of functional separation have found abnormal activity in the prefrontal cortex, limbic system, basal ganglia, and other local brain regions of patients with PD-D (Wen et al., 2013; Luo C. et al., 2014; Sheng et al., 2014; Hu et al., 2015a). Some studies using the method of seed point functional connectivity have revealed abnormal functional connectivity in the prefrontal-limbic neural circuit of patients with PD-D (Luo C. et al., 2014; Sheng et al., 2014; Hu et al., 2015b; Huang et al., 2015; Liang et al., 2016), but this method has many confounding factors, and the results often depend on the selection of seed region.

Independent component analysis (ICA) is a powerful blind-source data-driven approach that does not require a prior assumption of a seed region (Tessitore et al., 2019). ICA can effectively identify the brain function subsystems in the resting state, which not only can separate the components with neurophysiological significance but also separate the noise signals such as heartbeats and breathing to remove noise interference. Using ICA, Wei et al. (2017) study found that depressive symptoms in patients with PD were associated with functional connectivity abnormalities in the basal ganglia network, left frontoparietal control network, default network, and salience network. Our previous study using ICA found that patients with PD-D have abnormal functional connectivity within and between large-scale networks (Liao et al., 2021). However, previous studies did not analyze the differences between patients with PD-D with different depression severity. This study used the resting-state ICA method to explore the functional connectivity changes in the prefrontal cortex, limbic system, and basal ganglia regions in patients with different degrees of depression in PD.

## Materials and methods

### Study subjects

This study was approved by the Ethics Committee of the Second Xiangya Hospital of Central South University. Patients with PD were recruited from the Department of Neurology from December 2019 to December 2021, and the healthy controls were recruited from the community. All participants in the study signed written informed consent.

All patients with PD met the clinical diagnostic criteria for PD according to the 2015 edition of the International Society for Movement Disorders (MDS) (Halliday et al., 2015). Depression in patients was scored using the Beck Depression Inventory (BDI) and patients were divided into three groups: PDND (PD with no depression, BDI score < 10), PDMD (PD with mild depression, 10 ≤ BDI score ≤ 15), and PDMSD (PD with moderate and severe depression, BDI score > 15) (Smarr and Keefer, 2011). A total of 120 PD patients including 48 PDMSD, 34 PDMD, 38 PDND patients, and 54 sex- and age-matched healthy controls were recruited. According to the exclusion and inclusion criteria, 106 PD patients (35 PDND, 29 PDMD, and 42 PDMSD) and 50 HC were finally included.

The inclusion criteria of PD were as follows: (1) patients were right-handed; (2) patients were withdrawn from antiparkinsonian or antidepressant medicines for more than 12 h; and (3) patients had no cognitive impairment, which was evaluated by mini-mental state examination (MMSE), with the MMSE score not lower than the corresponding education level (the secondary school level < 24 points, the primary school level < 20 points, and the illiterate < 17 points).

The exclusion criteria of PD were as follows: (1) patients had contraindications for magnetic resonance examination or could not cooperate with the examination; (2) patients had a history of long-term alcohol or other drug abuse; (3) patients had Parkinson’s syndrome or Parkinson’s superimposition caused by other causes; (4) Patients had previous major neuropsychiatric diseases or intracranial lesions such as brain trauma, stroke, or brain tumors found in current MRI scan; (5) patients failed to cooperate in completing clinical consultations and questionnaire surveys; and (6) patients’ head movements were too large on magnetic resonance images (head movement larger than 2 mm) to affect data analysis.

The inclusion criteria of HC were as follows: (1) subjects with matched age, gender, years of education, and other basic information with PD; (2) subjects had no cognitive impairment; (3) subjects were right-handed; and (4) subjects had no mental illness or major organic disease history.

The exclusion criteria of HC were as follows: (1) subjects had contraindications for magnetic resonance examination or could not cooperate with the examination; (2) subjects had a history of long-term alcohol or other drug abuse; (3) subjects had Parkinson’s syndrome or Parkinson’s superimposition caused by other causes; (4) subjects had intracranial lesions found by MRI scans; (5) subjects failed to cooperate with the completion of clinical inquiries and questionnaires; and (6) subjects’ head movements were larger than 2 mm.

### Clinical and neuropsychological assessment

All patients with PD underwent a detailed examination consisting of neurological physicals and questionnaire assessments by a neurologist, using the Hoehn–Yahr (H&Y) grading scale and the Unified Parkinson’s Rating Scale Part III (UPDRS-III). Depressive disorders were diagnosed by a psychiatrist in patients with PD-D based on the fifth edition of the Statistical Manual of Mental Disorders and Psychiatry (DSM-V). The severity of depression was assessed using scores from the Beck Depression Inventory (BDI, a self-rated scale) and the Hamilton Depression Rating Scale (HDRS, a clinician-administered depression assessment scale) (Hamilton et al., 2015). At the same time, the MMSE scale was used to detect the cognitive impairment of all subjects, and the subjects with obvious impairment of cognitive function were excluded. In addition, the patient’s age, gender, handedness, education level, disease course, and clinical symptoms were recorded before the MRI data acquisition.

### Magnetic resonance data acquisition

The MRI data of all subjects were collected with a 3T Skyra MRI scanner with a standard operation guideline. The subjects were asked to close their eyes and keep silent during scanning, but not fall asleep. The resting state scan procedure lasted for 508 s. The subjects’ resting-state function and T1-weighted structural image data were collected. Resting-state data were acquired using echoplanar imaging (EPI) sequences with the following specific parameters: repetition time (TR) = 2,500 ms, echo time (TE) = 25 ms, numbers of slices = 39, slice thickness = 3.5 mm, gap = 0 mm, voxel size = 3.75 mm × 3.75 mm × 3.75 mm, flip angle = 90°, field of view (FOV) = 240 mm, matrix = 64 × 64, and image data of 200 time points (volume = 200) were acquired by scanning.

T1-weighted structural image data were acquired using magnetization-prepared rapid gradient echo sequence (MPRAGE) acquisition. The specific parameters were as follows: TR = 1900 ms, TE = 2.01 ms, number of slices = 176, slice thickness = 1.0 mm, voxel size = 1.0 mm × 1.0 mm × 1.0 mm, Flip Angle = 9°, Field of View = 256 mm, and Matrix = 256 × 256. The T1-weighted structural scan procedure lasted for 488 s.

### Image data processing

#### Data preprocessing

Data were preprocessed on the MATLAB2014a platform using the resting-state functional MRI data analysis and processing toolkit REST.^1^ It mainly included the following nine steps: (1) performing data format conversion (DICOM to NIFTI); (2) removing the data of the first 10 time points to exclude unstable machine signals, subjects’ incompatibility with the environment, and the influence of the drift of the magnetic resonance instrument caused by the instability of the magnetic field signal on the experimental results; (3) performing slice timing: aligning the scan times of all slices of the whole brain to the reference slice (the middle slice of the scan sequence). (4) performing head movement correction (realign): subjects with head movement exceeding 2 mm or rotation exceeding 2° were excluded; (5) normalizing (spatial normalization): performing standard brain analysis on all subjects based on T1-weighted structural phase parallelized Montreal Neurological Institute (MNI) functional image template spatial normalization; (6) performing smooth: taking 6 mm as the full width at half maximum (FWHM) to perform Gaussian processing for spatial smoothing on the data to reduce the random signal ratio of magnetic resonance; (7) detrending: removing the deviation of the image data from the physiological baseline caused by the subject’s head movement, heartbeat, and breathing during the data acquisition process; (8) removing covariates (Nuisance covariates and regression): removing covariates such as white matter, cerebrospinal fluid, 24 head movement parameters, and whole-brain signals; and (9) performing filter (Filer): selecting 0.01 to 0.08 HZ as the filter frequency band.

#### Data post-processing

After the data were preprocessed, the resting-state functional images were analyzed by independent component analysis using the resting-state data processing package GIFT^2^ on MATLAB2014a. The data analysis process mainly included four steps: dimensionality reduction, group ICA, post-reconstruction, and matching network of interest and independent components. (1) Dimensionality reduction of resting state data: dimensionality reduction of data was set to principal component analysis (PCA); (2) group ICA: The ICA algorithm (Infomax) was used to calculate the spatially independent components of the dimensionally reduced data, and the number of components was automatically estimated by the ICA algorithm (the automatic estimated value was 77). Independent components were evaluated for reliability using the ICASSO toolkit with 20 replicates; (3) post-reconstruction: reconstructed by the GICA algorithm based on the composition of each subject. Each reconstructed component contained the spatial Z-value image and the corresponding time series; and (4) matching the network of interest with independent components: The network components were selected by template matching principle and visual inspection. The template was Yeo template.^3^ Finally, nine components were selected, including anterior default mode network (aDMN), superior-posterior default mode network (spDMN), inferior-posterior default mode network (dDMN), left frontoparietal network (LFPN), right frontoparietal network (RFPN), precuneus network (PREN), interior salience network (inSN), basal ganglia network (BGN), and lateral salience network (laSN). These components covered the functional connectivity networks in brain regions related to the prefrontal, limbic, and basal ganglia.

### Statistical analysis

#### Clinical characteristics

Statistical analysis of clinical variables and demographic-related data of the samples was performed using SPSS 23.0 (IBM, United States) statistical software package. One-way analysis of variance (ANOVA) and *post-hoc* two-sample *t*-test were used to compare measurement data between groups; Pearson chi-square test was used to compare group differences in measurement data; Wilcoxon rank sum test was used to calculate rank data. A statistical level of *P* < 0.05 was considered significant.

#### Analysis of functional connectivity within the network

The single-sample t-test of SPM12 software was used to evaluate the spatial map of each network, and the spatial distribution map of the interest network for each subject was obtained separately (Alphasim, *P* < 0.001). The one-way ANOVA (one-tailed) of SPM12 and *post-hoc t*-test (two-tailed) were then used to calculate group differences in functional connectivity within the network (*P* < 0.05, voxel size > 5). The correlations between functional connectivity within the network and HDRS score, BDI score, and UPDRS-III score were obtained by Pearson correlation analysis, and a *P* < 0.05 was considered significant.

## Results

### Demographic and clinical characteristics

There were no significant differences in age, gender, MMSE score, and years of education among the four groups that were observed (*p* > 0.05). The *post-hoc* test showed that the HDRS scores and BDI scores in the PDMSD and PDMD groups were significantly higher than in the PDND group; the HDRS score and BDI score in the PD-D group were significantly higher than in the HC group. There were differences in the UPDR-III scores among the PDMSD, PDMD, and PDND groups (*p* < 0.05). The *post-hoc* test found that the UPDR-III scores in the PDMSD and PDMD groups were significantly higher than in the PDND group (*P* < 0.001) (Table 1).

**TABLE 1:** Clinical variables and demographic-related information (mean ± SD).

HC (*N* = 50)		PDND (*N* = 35)	PDMD (*N* = 29)	PDMSD (*N* = 42)	Statistic	*p*
Gender (M/F)	22/28	20/15	19/10	23/19	Pearson’s chi-squared	0.349
Age	55.420 ± 7.693	55.400 ± 9.86	55.621 ± 10.749	58.143 ± 9.619	ANOVA	0.471
Disease Duration (year)	NA	2.11 ± 2.11	1.97 ± 1.62	2.89 ± 2.24	ANOVA	0.11
Education (year)	8.23 ± 2.91	7.65 ± 3.24	8.04 ± 3.38	6.56 ± 2.67	ANOVA	0.058
MMSE score	27.47 ± 2.54	27.08 ± 3.35	26.67 ± 2.78	26.57 ± 2.51	ANOVA	0.33
HDRS- 17 score	2.68 ± 2.63	3.68 ± 3.57	12.28 ± 5.92	12.28 ± 5.92	ANOVA	0.00
BDI-21 score	9.18 ± 9.51	4.33 ± 3.32	12.40 ± 1.79	24.50 ± 9.64	ANOVA	0.00
UPDR-III score	NA	11.52 ± 7.63	20.08 ± 8.27	30.35 ± 8.69	ANOVA	0.00
H&Y score	NA	1.54 ± 0.63	1.65 ± 0.76	1.82 ± 0.66	Wilcoxon rank rum test	

HC, normal control; PDND, Parkinson’s disease without depression; PDMD, Parkinson’s disease with mild depression; PDMSD, Parkinson’s disease with moderate to severe depression; MMSE, Mini-Mental State Examination Scale; HDRS-17, 17 items Hamilton Depression Inventory; BDI-21, 21-item Baker Depression Inventory; UPDR-III, Parkinson’s Unified Rating Scale Part III; H&Y Rating, Hoehn–Yahr Rating Scale. Statistical analysis was performed using SPSS 23.0 statistical software.

### Identification of resting state networks

**FIGURE 1:**
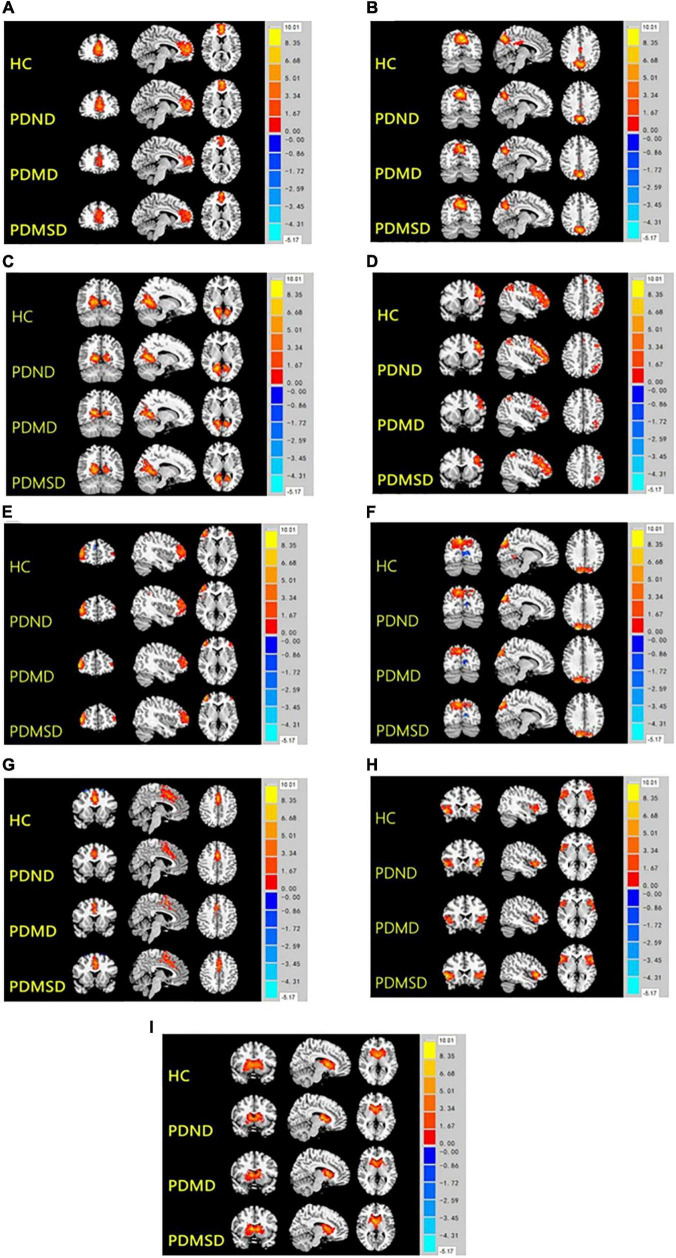
Nine spatial distribution maps of the intrinsic networks. HC, health control; PDND, PD with no depression; PDMD, PD with mild depression; PDMSD, PD with moderate and severe depression. 1A-1I, aDMN, ipDMN, spDMD, LFPN, RFPN, PREN, inSN, laSN, and BGN.

Nine spatial distribution maps of the intrinsic networks (one-sample t-test, *P* < 0.01, Alphasim, Figure 1) and the networks were selected from 77 components, including anterior default segment network (aDMN): medial prefrontal cortex (Figure 1A); posterior superior default network (ipDMN): medial temporal cortex and lateral parietal lobe (Figure 1B); posterior lower default network (spDMD): posterior cingulate (Figure 1C); left/right frontoparietal control network (LFPN/RFPN): left/right posterior parietal cortex (Figure 1D) and left/right lateral prefrontal cortex (Figure 1E); precuneus network (PREN): precuneus (Figure 1F); medial prominent network (inSN): anterior cingulate and some subcutaneous border structures (Figure 1G); lateral salience network (laSN): anterior insula and some subcutaneous marginal structures (Figure 1H); and basal ganglia network (BGN): putamen, caudate, globus pallidus, and thalamus (Figure 1I).

### Intergroup differences in functional connectivity within the network

Significant differences in functional connections were observed between the PDMSD, PDMD, PDND, and HC groups within the intra-network, including the anterior default network, the posterior upper default network, the posterior lower default network, the left frontoparietal control network, the right frontoparietal control network, the medial salience network, and the lateral salience network. Differences mainly appeared in the brain areas of the left precentral gyrus, right precuneus, right inferior frontal gyrus, right medial and paracingulate gyrus, left supplementary motor area, right insula, and left inferior frontal gyrus of the orbit (ANOVA, *P* < 0.05, Voxel size > 5, one-tailed) (Table 2).

**TABLE 2:** Brain regions with differences in functional connectivity within the network.

Network	Brain area (ALL)	Size	MNI coordinate			*F* value
			X	Y	Z	
aDMN (IC70)	Precentral L	11	–54	9	39	6.8035
spDMN (IC49)	Precuneus R_	9	9	–69	45	4.2018
ipDMN (IC20)	Precuneus R_	11	9	–72	33	5.4914
LFPN (IC70)	Precentral L	11	–54	9	39	6.8035
RFPN (IC29)	Frontal Inf Tri R	12	51	36	12	4.6482
InSN (IC66)	Cingulum_Mid_R	5	3	12	39	3.8747
	Supp_Motor_Area_L	5	–3	6	42	4.0153
laSN (IC35)	Insula R	9	39	9	–6	4.0153
	Frontal_Inf_Oper_L	9	–48	3	3	4.7659

ANOVA, *P* < 0.05, Voxel size > 5, one-tailed. AAL (Anatomical Automatic Labeling, provided by Montreal Neurological Institute). aDMN, anterior default node network; spDMD, posterior lower default network; ipDMN, posterior upper default network; LFPN, left frontoparietal network; RFPN, right frontoparietal network; inSN, medial salience network; laSN, lateral salience network; L, left side; R, Right. Precentral, precentral gyrus; Precuneus, precuneus; Frontal_Inf_Tri, deltoid inferior frontal gyrus; Cingulum_Mid, medial and paracingulate gyrus; Supp_Motor_Area, supplementary motor area; Insula, insula; Frontal_Inf_Oper, orbital inferior frontal gyrus.

### *Post-hoc* comparison of functional connectivity differences within the network

**FIGURE 2:**
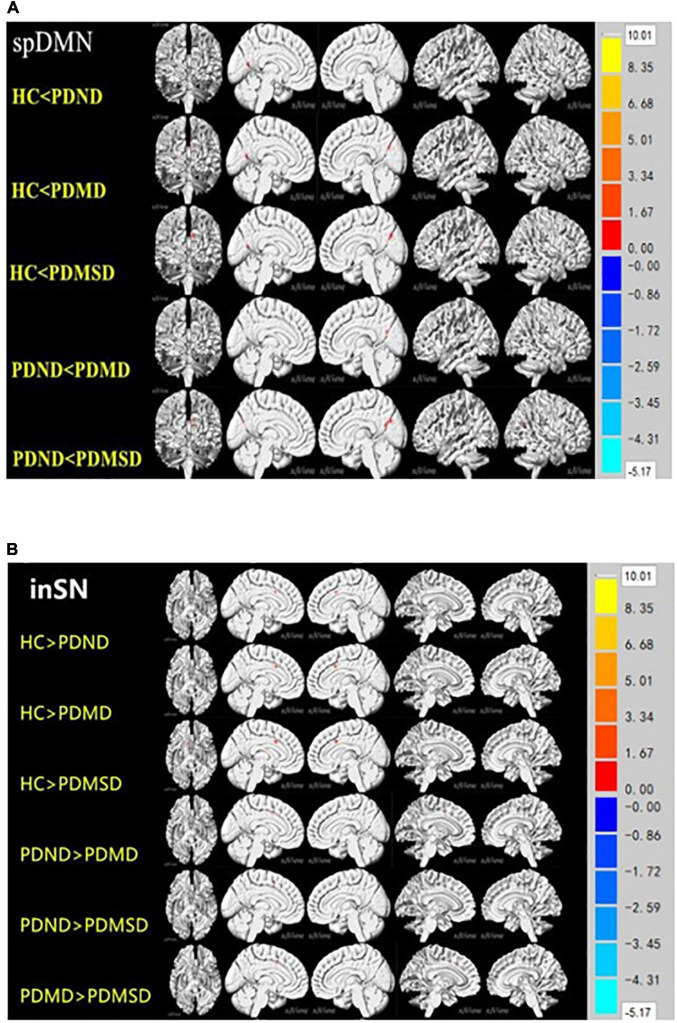
**(A)** Pairwise comparison results of the lower default network (spDMN) at the rear. *P* < 0.05, voxel zise > 5; HC: health control group; PDND: Parkinson’s disease without depression group; PDMD: Parkinson’s disease with mild depression group; PDMSD: Parkinson’s disease with moderate to severe depression group. Compared with the HC group, the functional connectivity of the PDND group in the cortical network around the left calcarine fissure was enhanced; the PDMD and PDMSD groups had enhanced functional connectivity in the cortical network around the bilateral calcarine fissure; compared with the PDND group, the PDMD and PDMSD groups had enhanced functional connectivity The group had strong functional connectivity within the right calcarine network. **(B)** Inner salience network (inSN) pairwise comparison results. *P* < 0.05, Voxel size > 4. The functional connectivity in the bilateral limbic lobe of the inSN was decreased in the HC group compared with the PDND group, PDMD group, and PDMSD group. Functional connectivity within the bilateral limbic lobe network was decreased in the PDMD group, the PDMSD group, and in the inSN compared with the PDND group.

The ROIs of the different brain regions were extracted and used as the Mask for further pairwise comparison (*P* < 0.05, Voxel size > 5). The functional connectivity was increased in the spDMN network, but it was decreased in the LFPN and inSN networks in the PDND group, PDMD group, and PDMSD groups when compared with the HC group. The functional connection was also increased in the spDMN network, whereas it was decreased in the LFPN and inSN networks in the PDMD and PDMSD groups when compared with the PDND group (Figure 2A); however, there was no significant difference between the PDMD and PDMSD groups. The functional connectivity in the inSN network was decreased in the PDMSD group when compared with the PDMD group, but there was no statistical difference in the functional connectivity in the spDMN and LFPN networks between the PDMSD group and PDMD (Figure 2B). *Post-hoc* comparisons showed no significant difference in functional connectivity within the aDMN, ipDMN, laSN, and RFPN networks between all groups.

### Correlation analysis between functional connectivity and Unified Parkinson’s Rating Scale Part III, Hamilton Depression Rating Scale, and beck depression inventory scores

The brain regions with significant differences between the PDMD and PDMSD groups and the PDND group were extracted. Pearson correlation was performed between functional connectivity changes in the corresponding networks and PD patients’ UPDRS-III, HDRS, and BDI scores. No statistically significant correlation was observed.

## Discussion

This study analyzed the functional connectivity changes in the networks of patients with PD with different degrees of depression using an independent component analysis method and explored the role of the neural network in the occurrence and development of depression in patients with PD. To our knowledge, this is the first study that attempted to explore alterations at the system or network level among patients with PD with different degrees of depression using ICA. This study found that impaired default network function is an important neurobiological basis for patients with PD with depression, dysfunction in the frontoparietal control network may be an imaging marker of PD-D, and dysfunction in the salience network may be an imaging marker for judging the severity of PD with depression.

### Abnormal default-mode network function is an important neurobiological basis for depression in Parkinson’s disease

The default mode network is mainly composed of the medial prefrontal lobe, posterior cingulate gyrus, precuneus, inferior parietal lobule, hippocampus, etc., which are active in the resting state and continuously negatively activated in the task state to regulate cognitive and emotional processing (Raichle et al., 2001). It is widely accepted that abnormal DMN function is an important neurobiological mechanism of depression (Raichle et al., 2001); however, the involvement of DMN in patients with PD with depression is still unclear. Lou et al.’s study comparing the brain functional connectivity between PD-D and PDND patients found that the functional connectivity of the right posterior cingulate gyrus was increased in PD-D patients, which significantly negatively correlated with depression severity (Lou et al., 2015). Hu et al.’s study found increased functional connectivity between the left medial cingulate gyrus and multiple major nodes in the DMN in patients with PD-D, which is associated with a decreased ability to self-regulation of emotions and increased sensitivity to negative emotional stimuli (Hu et al., 2015a). In contrast, Wei et al. found that patients with PD-D had decreased functional connectivity within the DMN (Wei et al., 2017). Consistent with our previous study (Liao et al., 2021) and other studies, this study found increased functional connectivity within the spDMN network in both the PDMD and PDMSD groups when compared with those in the PDND group. Therefore, our study further confirmed that the default network function, no matter if the functional connectivity within the DMN network is increased or decreased, is an important neurobiological basis for the occurrence of depression in PD. However, our study found no distinction between PD with severe depression and PD with moderate depression. Currently, the mechanisms of DMN acting on depression in patients with PD remain unknown. Some reports proposed that the increased functional connectivity is caused by compensation (Licata et al., 2013; Hillary et al., 2015), while some studies suggest that both increased and decreased functional connectivities reflect disruption of functional connectivity and abnormal functional communication of neuronal information (Esposito et al., 2010; Pawlitzki et al., 2017). It is postulated that patients repeatedly think about their symptoms, focus their attention on negative feelings, and fall into a vicious circle of negative emotions (Nolen-Hoeksema, 1991; Belzung et al., 2015; Lou et al., 2015), making it impossible to mobilize correct cognition in the task state and make DMN “negative activation,” which is manifested as an increase in DMN functional connectivity (Lou et al., 2015).

### Dysfunction in the frontoparietal control network may be an imaging marker of depression in Parkinson’s disease

The frontoparietal control network mainly includes the posterior parietal cortex and the dorsolateral prefrontal cortex, which is mainly involved in top-down attention and emotion regulation. Kaiser et al. (2015) found that abnormal functional connectivity within the frontoparietal control network was associated with uncontrolled emotional regulation and cognitive control in patients with depression. Several studies reported a decreased connectivity in the bilateral dorsolateral prefrontal hemispheres in patients with PD-D (Wen et al., 2013; Zhu et al., 2016). This study found that the functional connectivity within the LFPN network was reduced in the PDMD and PDMSD groups compared with the PDND group. Previous studies have suggested that the dysfunction of the frontoparietal control network, especially the dorsolateral prefrontal lobe, maybe a specific brain imaging marker that identifies depression in patients with PD (Pifl et al., 2013; Wen et al., 2013; Lou et al., 2015; Tucker et al., 2019; Santos-García et al., 2021). Moreover, one study identified the dorsolateral prefrontal cortex as a potential therapeutic target in patients with PD-D (Pifl et al., 2013). However, in our study, the functional connections in the frontoparietal control network showed no differences between PD with different degrees of depression. Thus, the dysfunction in the frontoparietal control network may be an imaging marker of PD-D, but it cannot distinguish the severity of depression. This is also consistent with most recent studies (Santiago et al., 2017; Tysnes and Storstein, 2017; Liu et al., 2019). Although it may implicate that the clinical difference between the PD with mild depression and the PD with moderate to severe depression is not significant, it may also be caused by a small sample size that is not large enough to be grouped separately.

### Dysfunction in the salience network may be an imaging marker for judging the severity of Parkinson’s disease with depression

The salience network (SN) is involved in the key parts of salient stimulation (Wen et al., 2013) and abnormalities in this network can cause abnormal processing of salient stimuli, which in turn can affect the onset and persistence of depressive symptoms (Hamilton et al., 2013; Luo Y. et al., 2014). However, the involvement of SN in the depression of patients with PD is rarely reported. This study found that either PDMD or PDMSD have reduced functional connectivity within the inSN network when compared with the PDND group, while the PDMSD group had reduced functional connectivity within the inSN network when compared with the PDMD group. This study suggests that the occurrence of depression in PD may be related to the reduction of functional connectivity within the salience network, and the severity of depression correlates with the degree of decreased functional connectivity within this network. Thus, abnormal function in the inSN may be an imaging marker for judging the severity of depression in PD.

We acknowledge several limitations of this study. First, the number of selected components of the ICA model is 77, which was automatically estimated, and there was no clear calculation standard. The separated components do not completely match the GIFT’s template, so whether the ICA model achieves the best is unsure. Second, due to the small number of patients with PD with major depression (only nine cases) in this study, it is not enough to group them separately. In the follow-up study, we will continue to collect patients with PD with major depression and group them separately. Third, it should be noted that the correction method in this study is relatively loose, and our results did not survive the more stringent correction methods (FDR and FWE). Thus, these conclusions still need further research to confirm. Fourth, although patients with PD in our study had stopped anti-PD medicines and anti-depressants for at least 12 h during the off-period before the MR scan and neuropsychological testing, it is still impossible to rule out the potentially confounding effects of chronic medications on the experimental results. Finally, this study was a cross-sectional study and did not follow up on changes in functional connectivity within dynamic brain networks in patients with PD.

In conclusion, the functional connections of some brain networks in patients with PD with different degrees of depression were changed, the functional connections in the spDMN network were increased, the functional connections in the LFPN and inSN networks were reduced, and the degree of reduced functional connections in the inSN network was related to the degree of depression in PD-D. The above findings provide an explanation of the brain network mechanism of PD with different degrees of depression.

## Data availability statement

The raw data supporting the conclusions of this article will be made available by the authors, without undue reservation.

## Ethics statement

The studies involving human participants were reviewed and approved by the Ethics Committee of the Second Xiangya Hospital of Central South University. The patients/participants provided their written informed consent to participate in this study.

## Author contributions

HL, QL, and ZM contributed to the conception and design of the study. SC, QS, MW, JLi, LZ, FZ, CS, JY, and YL contributed to the data collection. QL, ZM, CT, and JLiu contributed to the data analysis. QL and ZM contributed to writing the manuscript. All authors contributed to the article and approved the submitted version.

## Footnotes

1
http://restfmri.net

2
http://mialab.mrn.org/software/gift/index.html

3
https://surfer.nmr.mgh.harvard.edu/fswiki/CorticalParcellation_Yeo2011
